# Association between Perceived Health-Related Quality of Life and Depression with Frailty in the FRASNET Study

**DOI:** 10.3390/ijerph192416776

**Published:** 2022-12-14

**Authors:** Giulia B. Delli Zotti, Lorena Citterio, Sara Farinone, Maria Pina Concas, Elena Brioni, Laura Zagato, Elisabetta Messaggio, Sipontina Faienza, Marco Simonini, Alessandra Napoli, Valentina Di Mattei, Patrizia Rovere-Querini, Lucio Sarno, Emilio Clementi, Angelo A. Manfredi, Chiara Lanzani, Paolo Manunta

**Affiliations:** 1 Clinical and Health Psychology Unit, IRCCS San Raffaele Scientific Institute, School of Psychology, Vita-Salute San Raffaele University, 20132 Milan, Italy; 2 Genomics of Renal Diseases and Hypertension Unit, IRCCS San Raffaele Scientific Institute, School of Nephrology, Vita-Salute San Raffaele University, 20132 Milan, Italy; 3 Institute for Maternal and Child Health, IRCCS “Burlo Garofolo”, 34137 Trieste, Italy; 4 Nephrology Operative Unit, IRCCS San Raffaele Scientific Institute, 20132 Milan, Italy; 5 Unit of Clinical Pharmacology, Department of Biomedical and Clinical Sciences, “Luigi Sacco” University Hospital, Università di Milano, 20122 Milan, Italy; 6 Division of Immunology, Transplantation and Infectious Diseases, IRCCS San Raffaele Scientific Institute, School of Medicine, Vita-Salute San Raffaele University, 20132 Milan, Italy; 7 Scientific Institute, IRCCS Eugenio Medea, 23842 Bosisio Parini, Italy

**Keywords:** frailty, aging, quality of life, inflammation, genetics, multidimensional model

## Abstract

Frailty is a major challenge facing the aging world. The phenotype of the frail subject is still far from being satisfactorily defined. We report data on mood, cognition, and quality of life (QoL) in relation to anamnestic factors, health, and socio-economic status in the FRASNET geriatric population (1204 subjects in stable health conditions), which is an observational cohort study that includes fairly balanced groups of Italian frail (421, 35%), pre-frail (449, 37.3%) and robust (334, 27.7%) subjects. A conditional inference tree analysis revealed a substantial influence of psychological variables on frailty. The physical indicator of QoL (Short Form Survey-36-Physical Component Summary, SF-36-PCS) was the predominant variable in the full model (threshold at 39.9, *p* < 0.001): higher frailty was found in subjects with a caregiver and lower SF-36-PCS. Frailty was also associated with the mental indicator of QoL (Short Form Survey-36-Mental Component Summary, SF-36-MCS), depression (Geriatric Depression Scale, GDS-15), leisure activities, and level of education. In support of the prominent role of inflammation in aging and mental illness, the SF-36-PCS score was correlated with the blood concentration of C-X-C motif chemokine ligand 10 (CXCL10) (r Pearson −0.355, *p* = 0.015), a critical signal in cell senescence and inflammaging, while the rs7567647 variant in *FN1* gene encoding a glycoprotein in the extracellular matrix was significantly associated with frailty in a multivariable model (*p* = 0.0006). The perception of health-related QoL and subclinical depression contribute to frailty. Their assessment could improve the identification of older patients at increased risk of adverse outcomes.

## 1. Introduction

Frailty is a common and complex age-related clinical condition in older people, and it represents one of the major challenges for the healthcare system in aging societies. In European countries, the overall estimate of frailty prevalence is 18% (95% CI, 15–21%), even if actual prevalence can fluctuate by setting and definition of frailty [1]. The frailty phenotype is conceptualized by Fried [2] as “a biologic syndrome of decreased reserve and resistance to stressors, resulting from cumulative declines across multiple physiologic systems, and causing vulnerability to adverse outcomes”, included weakness, poor endurance, exhaustion, slowness, and low physical activity.

More recently, other dimensions have been taken into consideration to understand frailty syndrome [3,4,5,6,7], with biological, functional, cognitive, psychological, nutritional, and the socio-economic dimension playing a role [8].

Psychological factors such as cognitive impairment and depression, and physical conditions such as sarcopenia, also play a role [9,10]. An association between psychological factors such as cognitive impairment and depression, and physical conditions such as sarcopenia, has been reported [9,11]. In addition, frailty and depression are important conditions affecting older adults, and their relationship resulted to be robust ([12,13]).

Growing evidence supports the role of chronic inflammation as a causative mechanism of both depression and frailty among older adults [14]. Elevated levels of inflammatory signals, reflecting the interplay of genetic susceptibility, gut permeability, microbiota composition, defective mitophagy leading to persistent oxidative stress, and contraction of the T-cell repertoire associated with chronic exposure to microbial and endogenous antigens are hallmarks of aging. This condition is in itself harmful, being in part responsible for the multimorbidity, disability, and frailty typical of the elderly population. Indeed, it has been suggested that inflammation may have been evolutionarily selected for benefits early in life, despite detrimental effects in old age when the effect of natural selection is no longer active (antagonistic pleiotropy) [15]. Of importance, the dysregulated inflammatory state of the elderly (also known as “inflammaging”) is a well-characterized risk factor for chronic conditions such as sarcopenia and depression [16,17] while genes and inflammatory pathways associated with depressive symptoms are being actively studied in the elderly. The findings revealing a link between inflammation, immune dysregulation, and accelerated cellular aging could be relevant to the association we describe [18].

Frailty is also linked to health-related quality of life (QoL): an increase in the frailty-related items caused a poor quality of life for all dimensions [19]. A lower prevalence of frailty in the older adults is associated with higher physical activity, better economic conditions, higher levels of cultural fruition, social and affective interactions [20].

Despite this accumulating knowledge, it is still unclear how each of these factors relates to each other or what their relative role is in causing frailty. Effective prevention and treatment of the elderly patients require a multifaceted approach. A holistic understanding of the basis of frailty would allow more effective interventional personalized strategies that target socio-psycho-physiologic systems. In this study we focused on frailty determinants using a multidimensional approach that included anamnestic factors, psychological aspects, overall health and socio-economic status, together with immunological biomarkers and genetic variants.

## 2. Materials and Methods

### 2.1. Study Design

The Frailty and Sarcopenia Network (FRASNET) study involved the recruitment of older adults carried out on a voluntary basis at recreational centers, cultural centers, retirement homes in Milan and Monza Brianza areas, at San Raffaele Scientific Institute in Milan, and the Cuggiono Hospital, nearby Milan, Italy (Table S1). All study participants were volunteers and signed informed consents for participation in the study for privacy and for the collection and storage of biological material. 

### 2.2. Participants

#### 2.2.1. Data Collection

**Figure 1:**
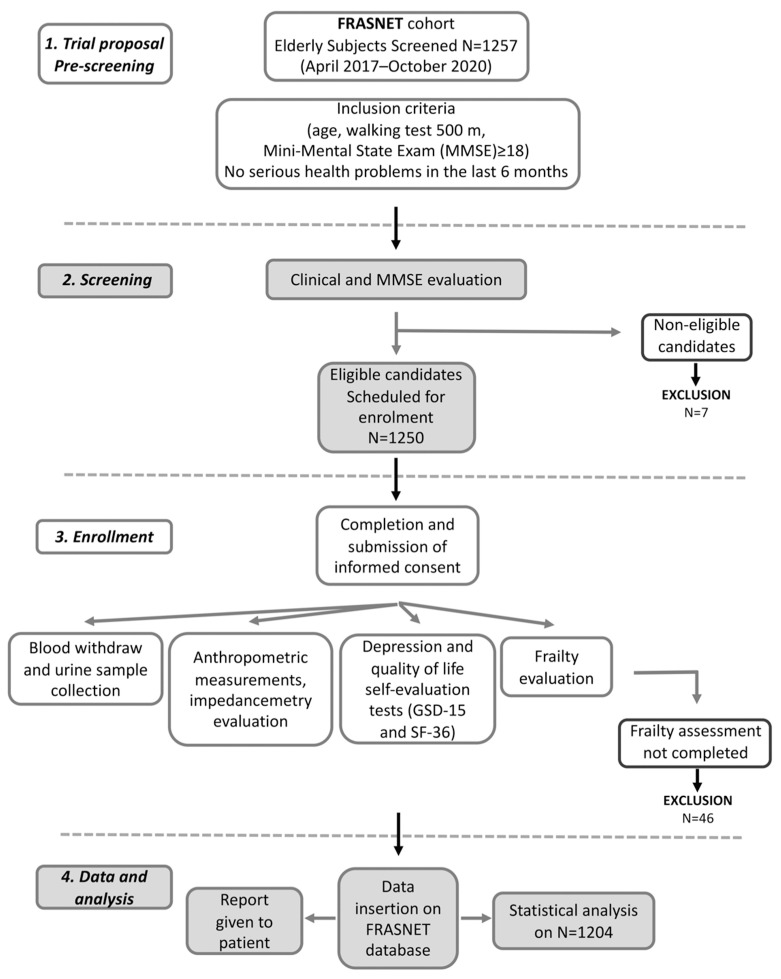
Flow chart for frailty analysis in FRASNET study.

FRASNET is a cross-sectional observational cohort study on 1250 older adults. The inclusion criteria were (1) age equal or of more than 65 years; (2) walking capacity more than 500 m without assistance (self-reported) [21]; (3) Mini-Mental State Examination (MMSE) [22] with score ≥ 18; and (4) absence of recent and severe health problems resulting in a life expectancy of fewer than 6 months. The exclusion criteria were: subjects not able to sign informed consent, severe health problems (e.g., uncontrolled hypertension, recent upper or lower extremity fractures, myocardial infarction within the past 1 year). The volunteers filled out a questionnaire for demographic and psychosocial data. Current pathologies and therapies have been recorded as well as information about falls and access to the emergency room relating to the former year. Anthropometric measurements were also performed; urine and blood samples were collected. Moreover, some questionnaires were submitted such as for QoL (the Short Form 36 (SF-36) Health Survey) [23], depression (the Geriatric Depression Scale (GDS-15)) [24,25], MMSE [22] for the psychological evaluation, and also for physical activity (Physical Activity Scale for Elderly (PASE) [26], and exhaustion (the Fatigue Severity Scale (FSS)) [27] for frailty determination. A flow chart for subjects screening, enrolment, and data management is reported in Figure 1. The data were collected and reported in an electronic case report form (eCRF) created using FileMaker Pro v. 11.

#### 2.2.2. Psychometric Measurements

##### Mini-Mental State Examination (MMSE) 

The MMSE [22] is a measure of cognitive impairment; it includes eleven questions of orientation, registration, recall, calculation and attention, naming, repetition, comprehension, reading, writing, and drawing, which provide a summed score of the current severity of cognitive impairment; the maximum total score is 30. 

Subjects with severe cognitive impairment (MMSE < 18) were excluded from the subsequent phases. The determination of the cut-off at 18 derives from the validation study of the tool, which stratified the cognitive impairment in the following ranges: 24–30 normal (specifically, scores in the range of 30–28 can be distinguished as indicative of subjects with “normal-high-end” cognitive functioning; scores in the range of 27–24 are instead indicative of “normal-low-end” cognitive functioning); while 18–23 indicate medium impairment; and 0–17 indicate severe impairment, which is a range not included in this study. Subsequently, all patients who do not have severe cognitive impairment (scores between 18 and 30 at the MMSE) were administered the GDS-15 and SF-36 tests, aimed, respectively, at evaluating the depressive state and the self-perceived QoL. 

##### Geriatric Depression Scale (GDS-15)

The GDS-15 is a self-report questionnaire consisting of 15 items, which has been validated for the assessment of depression in older adults [24,25]. Therefore, it taps the affective and behavioral symptoms of depression and excludes most symptoms that may be confused with somatic disease (e.g., slowness, insomnia, hyposexuality) or dementia. The 15-item version takes about 5−7 min to complete. Obtaining a score ≥6 on this scale is indicative of a depressive mood and is evaluated with the other clinical data of the patient. Any score >6 should be evaluated in conjunction with the patient’s other clinical data to obtain a better picture of the patient’s situation. 

##### SF-36 Medical Outcomes Study Questionnaire Short Form 36 (SF-36) Health Survey

SF-36 is a generic, self-administered, multidimensional questionnaire related to the QoL, with the aim of assessing the state of self-perceived psycho-physical health through 36 items, which were divided into 8 sub-scales yielding two summary measures: the physical component summary (Short Form Survey-36-Physical Component Summary (SF-36-PCS)) and the mental component summary (Short Form Survey-36-Mental Component Summary (SF-36-MCS)) (see Methods) [23].

All items have a response with a Likert scale score. It measures health on eight multi-item dimensions, covering functional status, well-being, and overall evaluation of health. The scores obtained from the test are weighted by gender and age and provide an index of physical (PCS) and mental (MCS) health. They are categorized according to the following modality: 71–100, indicative of psycho-emotional/psycho-physical well-being; 40–70, indicative of an average psycho-emotional/psycho-physical well-being; 31–39, indicative of poor psycho-emotional/psycho-physical well-being; 0–30, indicative of psycho-emotional/psycho-physical malaise [23]. 

#### 2.2.3. Physical Activity Scale for Elderly (PASE) and Fatigue Severity Scale (FSS)

PASE is a practical and widely questionnaire used for physical activity assessment in epidemiologic investigations. It is a brief and easily scored survey designed specifically to assess physical activity in studies of persons aged 65 years and older. The PASE score combines information on leisure, household, and occupational activity. It assesses the types of activities typically chosen by older adults (walking, recreational activities, exercise, housework, yard work, and caring for others). It uses the frequency, duration, and intensity level of activity over the previous week to assign a score, with higher scores indicating greater physical activity [26]. The FSS is designed to differentiate fatigue from clinical depression, since both share some of the same symptoms [27]. The FSS questionnaire contains nine statements that attempt to explore the severity of fatigue symptoms: a low value indicates that the statement is not very appropriate, whereas a high value indicates agreement.

#### 2.2.4. Frailty Definition and Assessment

To identify frail older adults, an adapted version of the frailty phenotype of Fried was used [2]. A four-item version of the Fried criteria was considered: (1) exhaustion, self-reported by the FSS questionnaire on carrying out daily activities; (2) low strength, evaluated by the test of the chair (getting up and sitting down from a chair five consecutive times without using upper limbs); (3) slow walking speed, evaluated by a ten-meter walking test, also with aids; and (4) low physical activity, established by a PASE questionnaire. The final score was calculated as the sum of the individual component scores (range 0–4). Subjects with three or more criteria are classified as frail, while those with one or two are classified as pre-frail, and those meeting none are classified as robust.

### 2.3. Laboratory Investigations

Simultaneous assessment of plasma concentrations of a series of twenty-five cytokines was performed on a subset of the FRASNET cohort, 75 samples selected among robust (n = 26), pre-frail (n = 12), and frail (n = 37) subjects, by using a commercially available multiplex bead-based sandwich immunoassay kit (Bio-Plex Pro Human Cytokine 27-Plex Immunoassay, Bio-Rad, Segrate (MI), Italy) [28], including interleukins (IL-1ß, IL-1 Receptor antagonist, IL-4, IL-6, IL-7, IL-8, IL-9, IL-10, IL-13, IL-17, IL-18), eotaxin, basic fibroblast growth factor (FGF), interferon-γ (IFN-γ), C-X-C motif chemokine ligand 10 (CXCL10), C-C motif chemokine ligand 2 (CCL2), C-C motif chemokine ligand 3 (CCL3), C-C motif chemokine ligand 4 (CCL4), C-C motif chemokine ligand 5 (CCL5), platelet-derived growth factor subunit B (PDGF-BB), and tumor necrosis factor-alpha (TNF-α). A range of 3–10,000 pg/mL of recombinant cytokines was used to establish standard curves. Cytokine levels were determined using a Bio-Rad BioPlex 200 by Bio-Plex Manager software 6.0 (Bio-Rad, Segrate (MI), Italy). In Table S2, descriptive statistics of all measured cytokines are reported.

Genotyping of targeted single nucleotide polymorphisms (SNP) was performed on genomic DNA from the entire cohort. Genomic DNA was extracted from peripheral whole blood automatically by Maxwell^®^ RSC Blood DNA (Promega, Madison, WI, USA). The genetic characterization of SNPs was carried out by the TaqMan OpenArray Genotyping System (Applied Biosystems, Foster City, CA, USA) with custom OpenArray plates designed for multiple SNPs (https://tools.thermofisher.com/content/sfs/manuals/MAN0014351_OpenArray_Genotyping_Experiments_QR.pdf; accessed on 2 July 2019). On the basis of previous genetic studies related to frailty [29,30], aging [31,32], and psychological parameters [33,34,35], 16 SNPs have been selected, as reported in Table S3. For analysis of the genotypes, auto-calling methods have been used as implemented in the TaqMan Genotyper software version 1.6. One hundred and thirty duplicated samples gave 100% reproducibility for all SNPs. The global genotyping reached a call rate equal to 97.4%.

### 2.4. Statistical Analysis

Continuous variables were tested for normal distribution by the Shapiro test; those that did not show to be normal were mathematically transformed in an appropriate way to restore normality (log or ranked values) or have been transformed into clinical classes. MMSE was analyzed as a binary parameter: 24–30 normal and 18–23 medium impairment. GDS-15 was analyzed as three classes: 0–5 normal score, 6–9 mild depression score, and 10–13 depression score [25]. Both SF-36-PCS and SF-36-MCS were mathematically transformed as ranked values to restore normal distribution. Data were represented as numbers and frequencies for the categorical variables, as mean ± SD for the canonical continuous variables and median, and quartiles for the non-normally distributed variables.

Different types of analyses were applied depending on the purpose. For the general relationship between the outcome variable (frailty) and each of the four psychological variables (SF-36-PCS, SF-36-MCS, GDS-15, MMSE), and for the identification of the frailty determining factors, nominal regression was executed (R library MASS, polr function). In these analyses, the age, sex, weight, number of daily drugs, presence of a caregiver, annual income, number of schooling years, and presence of leisure activities were introduced as covariates. Particularly, the analyses of MMSE as an outcome did not consider age and the level of education variables because they were already included in the standardization of its scoring, while the analyses of both health indexes of QoL (SF-36-PCS and SF-36-MCS) did not consider sex, age, and the presence of leisure activities variables because they were already included in the standardization of their scoring.

Cytokine continuous variables with non-normal distribution were mathematically transformed as log, and linear regression analysis was performed in relation to both the SF-36 psychological parameters, or univariate analysis was performed for the MMSE and GDS-15. SNP analysis was performed by univariate analysis for quantitative parameters as SF-36, by logistic regression for MMSE, and nominal regression for GDS-15 and frailty as dependent variables, with the appropriate covariates.

To determine the influence of the selected explanatory variables on the outcome variable (frailty) and for the construction of a global model, conditional inference tree analysis was carried out using the ctree function of R’s Partykit package (https://cran.r-project.org/web/packages/partykit/vignettes/partykit.pdf; accessed on 14 March 2022). This statistical method [36] performs recursive partitioning that allows the creation of a regression tree; the variable with the highest predictive power (the lowest *p* value after Bonferroni correction) is represented as the first node in the decision tree, so two subgroups (I and II) are created. For subgroup I, the variable with the lowest *p* value (if any) is taken as the second or third node. The same is completed for subgroup II. The final model is based on the subdivision variables in each node with the highest statistical significance. The branching stops when the divisions are not significant. Therefore, the regression tree shows the data broken down into smaller subsets that differ significantly in the level of the response variable. Nodes and branches are represented according to the hierarchical order of the predictive power of the relative explanatory variable. Therefore, the higher the position of the node in the tree, the more powerful the influence of the variable on the variability of the response. Conditional inference tree analysis was also performed by testing the influence of each SNP, whose result was significant in the previous regression analysis. Statistical analysis was carried out using SPSS v.21 software or R package v. 4.1.0.

## 3. Results 

### 3.1. Baseline Characteristics of Participants

**Figure 2:**
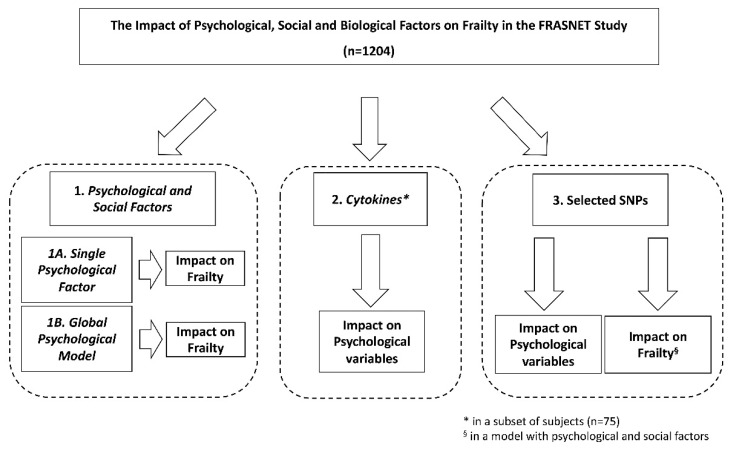
Schematic representation of the analyses performed on the recruited FRASNET cohort. SNPs = single nucleotide polymorphisms.

The FRASNET study enrolled a total of 1250 participants; of these, 1204 participants completed the frailty assessment and were eligible after the exclusion criteria (Figure 1) were recruited. The mean age was 73.29 ± 5.79 years; 40% were male and 60% were female. The sex of participants was defined based on both an external examination of body characteristics during enrolment and through genetic testing during the genotyping step. Frailty was found in 421 (35%) individuals of the studied population, while 449 (37.3%) were classified as pre-frail and 334 (27.7%) were classified as robust. Table 1 shows the descriptive statistics of the analyzed cohort, describing the social condition, health, and psychological status of volunteers. A schematic representation of the analyses performed on the recruited cohort and reported below is shown in Figure 2.

### 3.2. Impact of the Physical Health Measure of Quality-of-Life Model (SF-36-PCS) on Frailty

Frailty was first analyzed using a nominal regression model taking into consideration SF-36-PCS, BMI, daily drugs, presence or absence of the caregiver, annual income, and level of education as predictor variables. The regression resulted in a statistical significance of the model (*p* < 0.001) and of SF-36-PCS (OR 0.51, 95% confidence interval (CI) 0.44–0.57, *p* < 0.001), but also of the number of daily drugs (OR 1.24, 95% CI 1.11–1.39, *p* < 0.001) and level of education (OR 0.85, 95% CI 0.75–1.56, *p* = 0.0065); see Table S4.

**Figure 3:**
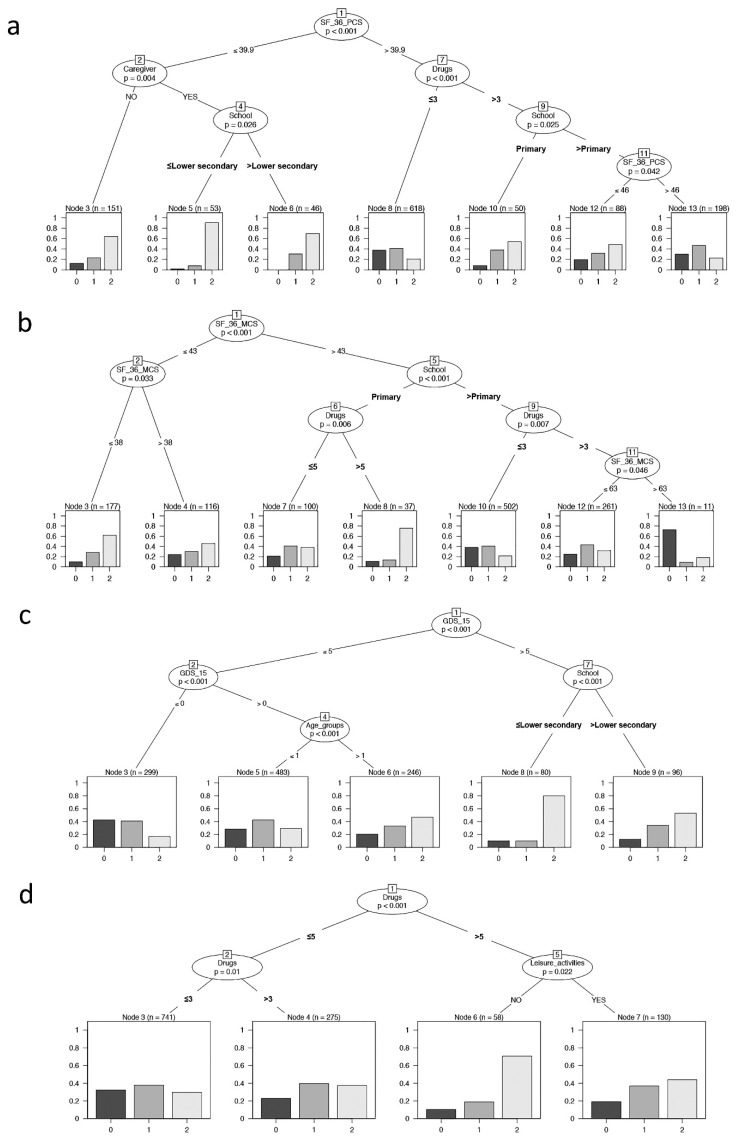
Binary tree computed by conditional recursive partitioning by psychological parameters. The terminal nodes showed the direct impact of these variables on frailty. (**a**) Effect of SF-36-PCS (the physical indicator of health-related quality of life), together with BMI classes, number of daily drugs (in quartile, “Drugs”), absence or presence of caregiver (NO/YES), annual income, and level of education (School), on frailty classes (0 = robust, 1 = pre-frail, 2 = frail subjects). The variables affecting frailty are SF-36-PCS, having or not having a caregiver, education level, and the number of daily drugs. (**b**) Effect of SF-36-MCS (the mental indicator of the quality of life), together with BMI classes, number of daily drugs (“Drugs”), absence or presence of caregiver (NO/YES), annual income, and level of education (School), on frailty classes (0 = robust, 1 = pre-frail, 2 = frail subjects). The variables affecting frailty are SF-36-MCS, level of education, and the number of daily drugs. (**c**) Effect of GDS-15 (0–5 = no depression, 6–9 = mild depression, 10–13 = depression), together with sex, age groups, BMI classes, number of daily drugs (“Drugs”), absence or presence of caregiver (NO/YES), annual income, absence or presence of leisure activities (NO/YES), and level of education (School), on frailty classes (0 = robust, 1 = pre-frail, 2 = frail subjects). The variables affecting frailty are GDS-15, level of education, and age groups. (**d**) Effect of MMSE, together with sex, age groups, BMI classes, number of daily drugs (“Drugs”), absence or presence of caregiver (NO/YES), annual income, and absence or presence of leisure activities (NO/YES), on frailty classes (0 = robust, 1 = pre-frail, 2 = frail subjects). The variables affecting frailty are the number of daily drugs and leisure activities.

The conditional inference tree analysis (Figure 3a) showed that SF-36-PCS is the first variable affecting frailty (node 1: SF-36-PCS = 39.9 as cut-off, *p* < 0.001). In addition, having or not having a caregiver, daily drugs, and level of education significantly influenced the outcome. The terminal nodes showed the distribution of frailty in each identified subsample (Node 3, 5, 6, 8, 10, 12,13). Nodes 3, 5, and 6 represent individuals with SF-36-PCS ≤ 39.9 and denoted individuals with the highest percentages of frailty. In particular, node 5 identified an increased percentage of frail individuals: these subjects had the presence of a caregiver and a level of education of lower secondary school or less. On the contrary, node 8 considered a higher proportion of robust individuals and are those with a lower number of drug administration and with SF-36-PCS > 39.9. Finally, considering individuals with SF-36-PCS > 39.9, node 10 showed an increased percentage of frailty in subjects with a lower level of education, while node 13 showed the highest level of pre-frail and represented the subset of individuals with both a higher level of education and of SF-36-PCS.

### 3.3. Impact of the Mental Health Measure of Quality-of-Life Model (SF-36-MCS) on Frailty

When the predictor variables included SF-36-MCS, BMI, number of daily drugs, presence or absence of the caregiver, annual income, and level of education in relation to frailty, the nominal regression resulted in a statistical significance of the model (*p* < 0.001) and of SF-36-MCS (OR 0.63, 95% CI 0.56–0.71, *p* < 0.001) but also of the number of daily drugs (OR 1.36, 95% CI 1.22–1.52, *p* < 0.001) and level of education (OR 0.80, 95% CI 0.71–0.90, *p* < 0.001), (Table S5).

Similarly, in the conditional inference tree analysis SF-36-MCS (Figure 3b), the level of education and daily drugs significantly influenced the frailty; in particular, SF-36-MCS was the first variable that affected frailty (node 1: SF-36-MCS = 43 as cut-off, *p* <0.001). The total partition in seven final subgroups showed that the higher proportion of frail individuals included both those with a lower level of SF-36-MCS (node 3) or a lower educational level and the higher number of drug administration despite a good SF-36-MCS performance (node 8). Alternatively, node 10 identified the higher proportion of robust and pre-frail individuals that fell in groups having higher levels of SF-36-MCS, school education, and lower number of daily drug administration.

### 3.4. Impact of Depression Model (GDS-15) on Frailty

In the nominal regression, the predictor variables included depression (GDS-15) expressed in three classes, in addition to sex, age, BMI, number of daily drugs, presence or absence of the caregiver, annual income, level of education, and leisure activities. The regression resulted in a statistical significance of the model (*p* < 0.001) and of GDS-15 (OR 2.51, 95% CI 1.95–3.28, *p* < 0.001) but also of age (OR 1.58, 95% CI 1.26–1.99, *p* < 0.001), number of drugs (OR 1.24, 95% CI 1.11–1.39, *p* < 0.001), level of education (OR 0.81, 95% CI 0.72–0.92, *p* < 0.001) and leisure activities (OR 0.60, 95% CI 0.46–0.78, *p* < 0.001), (Table S6).

As reported in Figure 3c, the binary tree showed that GDS-15, age groups, and level of education significantly influenced the frailty, with GDS-15 being the most influencing variable affecting frailty (node 1: GDS-15 = 5 as cut-off, *p* < 0.001). The resulting subgroups provided that from node 3 to nodes 8–9, there was an increasing percentage of pre-fail and fail individuals due to the increment of age for individuals without depression (GDS-15 < 5, nodes 5–6) and a marked increase in frail individuals with decrease in schooling years for individuals with depression (GDS-15 > 5, nodes 8–9).

### 3.5. Impact of the Cognitive Model (MMSE) on Frailty

When the MMSE, cognitive impairment expressed in classes, sex, BMI, number of daily drugs, presence or absence of the caregiver, annual income, and leisure activities were considered in relation to frailty, the regression resulted in a statistical significance of the model (*p* < 0.001) and of MMSE (OR 0.56, 95% CI 0.35–0.89, *p* = 0.014), but also of number of drugs (OR 1.39, 95% CI 1.25–1.55, *p* < 0.001) and leisure activities (OR 0.57, 95% CI 0.44–0.74, *p* < 0.001), (Table S7).

As reported in Figure 3d, the binary tree showed that only daily drugs and leisure activities significantly influenced frailty. This context showed that the higher proportion of frail individuals was that with a higher number of drug administration and without leisure activities (node 6), while the most robust group was retrieved from those with a lower number of daily drugs (node 3).

### 3.6. Global Psychological Model on Frailty

**Figure 4:**
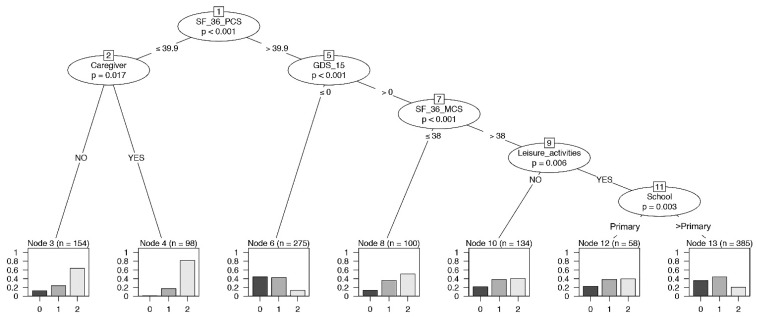
Binary tree computed by conditional recursive partitioning on frailty classes, global model. Effect of SF-36-PCS (the physical indicator of health-related quality of life), SF-36-MCS (the mental indicator of the quality of life), GDS-15 (0–5 = no depression, 6–9 = mild depression, 10–13 = depression), MMSE, together with sex, age groups, BMI classes, number of daily drugs (“Drugs”), absence or presence of caregiver (NO/YES), annual income, absence or presence of leisure activities (NO/YES), and level of education (School), on frailty classes (0 = robust, 1 = pre-frail, 2 = frail subjects). The variables affecting frailty are SF-36-PCS, having or not having a caregiver, GDS-15, SF-36-MCS, leisure activities, and education.

A conditional inference tree analysis for frailty was performed simultaneously including all the psychological variables as predictors. SF-36-PCS, caregiver, GDS-15, SF-36-MCS, leisure activities, and educational level significantly influenced the frailty (Figure 4). This model allowed us to focus on the important role of the variables SF-36-PCS, SF-36-MCS, and GDS-15 among the four psychological parameters. The conditional inference tree identified the highest proportion of robust individuals in the group with a higher performance of SF-36-PCS and no depression status (node 6). On the contrary, node 4 displayed the highest level of frail individuals with a lower SF-36-PCS level and the presence of a caregiver. In addition, a lower SF-36-MCS in the context of a depression status (>0) reported a high proportion of pre-frail and frail individuals (node 8).

### 3.7. Immunological Determinations

The levels of all measured cytokines in a subset of subjects were analyzed in relation to the four psychological parameters as both nominal and covaried by sex, age, and BMI analyses: MMSE and GDS-15 were studied using univariate analyses, while SF-36-PCS and SF-36-MCS were studied using linear regression. Levels of CXCL10, which has been proposed to represent an evolutionarily conserved marker of inflammaging and frailty [37], were selectively and significantly inversely correlated with the SF-36-PCS (r Pearson −0.355, *p* = 0.015 (model) and *p* = 0.015 covaried; *p* = 0.003 nominal). 

### 3.8. SNPs and Psychological Parameters

Genetic variants in Table S3 were tested for their association with the psychological parameters above mentioned and with frailty. A schematic summary of association results is reported in Table 2. After pruning by the FDR multiple test comparison, some significant SNPs were found associated with SF-36-PCS (*CNTF* rs1800169, *TGFB1* rs1800469, *MTR* rs1050993), with MMSE (*IL6* rs1800795), and with frailty (*FN1* rs7567647), while no associations were found with SF-36-MCS and GDS-15. None of these SNPs was associated with more than one dependent variable.

When these genetic variants were included in the conditional inference tree analysis in which frailty was the response variable and the psychological parameters were explanatory variables, only *FN1* rs7567647 displayed a significant association (Figure S1). Briefly, we observed that in the subgroup of individuals with SF-36-MCS > 38 and SF-36-PCS ≤ 49, the SNP divided individuals carrying the AA genotype from those carrying AG or GG (node 8): the percentage of individuals with frailty class one and two was higher in the group of AA genotype carriers compared with the AG/GG carriers.

## 4. Discussion

In this study, we aimed at clarifying the complex relationship responsible for frailty condition using a multidimensional approach focused on psychological aspects (mood, cognition, health-related QoL) in relation to anamnestic factors, health, and socio-economic status. Our results reveal a strong impact of psychological variables on frailty in a population of relatively healthy Italian older adults. Indeed, SF-36-PCS highlighted the influence of psychological factors on frailty, which appeared at least as determinant as caregivers, daily medications, and levels of education, with lower SF-36-PCS scores (<39.9) and the presence of caregiver representing critical factors in particular in subjects with a lower level of education. For the mental QoL, SF-36-MCS also mostly impacted frailty jointly with daily medications, and level of education, with the model reporting the highest proportion of frail individuals among those with lower levels of SF-36-MCS (≤43).

Previous studies examined the relationship between frailty and psychological variables [38,39,40,41] but separately. Focusing on depression, GDS-15 prevailed over the entire model in frailty impact, which is flanked by the level of education and age (leisure activities and the number of medications only in regression). A GDS-15 score of no depression corresponded to higher percentages of robust and pre-frail subjects; higher percentages of frailty, otherwise, were found in individuals with lower education levels and depression. This well agrees with the results of Ji and colleagues [42]. Alternatively, with respect to MMSE cognitive status, the binary tree indicated that only the number of daily drugs, considered an index of comorbidity [43], and leisure activities significantly influenced the frailty status with any impact of MMSE itself, showing the higher proportion of frail individuals among those with a higher number of drugs and without leisure activities, while the most robust group was retrieved from those with a lower number of daily drugs. The whole regression analysis reported a significant association also for MMSE, and a recent review also suggests a bidirectional relationship between frailty and cognitive impairment [44]. The apparent discordance may be justified by the inclusion criteria of MMSE ≥18 in our study.

So far, results on frailty at multilevel analysis have not been reported yet. Therefore, an overall psychological model emerging from the conditional inference tree analysis was here examined. In addition, this analysis identifies SF-36-PCS as the predominant variable in the entire model, with the subgroup with higher SF-36-PCS performance and no depression status having the highest percentage of robust and pre-frail individuals. In contrast, the highest level of frail individuals showed a lower SF-36-PCS level (<39.9), without but especially with a caregiver. SF-36-MCS, GDS-15, leisure activities, and educational level also significantly influenced the frailty status. 

A subset of the FRASNET cohort was also characterized for the profile of inflammatory cytokines in relation to each of the four psychological parameters described above. A relatively large panel of twenty-five signals was considered. However, a single cytokine appeared informative in the FRASNET cohort, in the absence of existing neoplastic, infectious, or inflammatory comorbidities, with a significant inverse relationship between the SF-36-PCS score and levels of CXCL10. This finding agrees with the negative association reported in the MyoAge cohort between blood CXCL10 concentration and working memory performance in older subjects, which was possibly associated with age-related altered methylation within the CXCL10 gene promoter [45] and with the critical role of CXCL10 as a marker of a restricted repertoire of T cells in older subjects predicting a negative outcome of COVID-19 [28].

In the FRASNET cohort, the most informative SNP resulted in the rs7567647 for association with frailty and affecting the binary tree. The SNP is located in an intronic region of *FN1*, which is a gene that encodes for fibronectin 1, a glycoprotein in the extracellular matrix involved in cell adhesion and migration processes including embryogenesis, wound healing, blood coagulation, and host defense [46]. A previous study reported a big panel of polymorphisms related to inflammation and muscle maintenance, showing this *FN1* genetic variant in association with frailty [29]. The A allele exhibited an OR 4.20 (1.69–10.39) for frailty, and the same trend of association was found in the current FRASNET study. 

An extensive assessment in older adults to identify deterioration of the QoL (SF-36-MCS < 43; SF-36-PCS < 39.9), initial depressive symptoms (GDS-15 ≥ 5), together with anamnestic factors, health, and socio-economic conditions, as an early identification of the older adults at risk, might protect from the onset of frailty syndrome or its worsening [47,48,49], possibly reducing the costs associated with psychological rehabilitation and psychiatric hospitalizations. Our results also support a model in which frailty is related, within the geriatric population, to a continuous activation of innate immunity that culminates in a low-intensity but continuous inflammatory response. In this group of subjects, this maladaptive activation of the immune response, facilitated by the genetic background, is likely and probably influenced by lifestyle habits as well as environmental and cultural conditions [50,51]. Further multicentric prospective studies are needed to investigate whether the higher concentration of CXCL10 can help stratify otherwise healthy older patients for the risk of frailty and neuropsychological involvement.

For the first time, the multidimensional holistic model of frailty considering psychological variables related to frailty status simultaneously but also socio-economic (annual income, level of education) and biological variables (BMI, sex, age, drugs, cytokines, genetics) spotlighted the most influencing variables on frailty. The strength of the peculiar conditional inference tree analysis is the accurate relationships between all these elements, which are usually studied separately [38,52,53,54,55], thus evidencing the impact of different variables on frail, pre-frail, and robust status in the older general population.

This study has limitations. First, no gold standard measurement of frailty was utilized, as the best method of frailty assessment has not been determined yet, even if the prevalence evaluated in this study is in agreement with previous studies [56,57]. Many researchers prefer to use the Fried method [2] also to assess related outcomes just because it is based on objective measurements such as handgrip and gait speed, which are more helpful for comparison among studies. A modified version of the Fried criteria is a critical point. We came to the decision to use a modified version of the criteria in which changes in body weight are not considered. The FRASNET study is a cross-sectional observational cohort study, and data and parameters were collected in a single visit, resulting in an assessment of a weight loss/increase necessarily drawn from a baseline self-report. Unfortunately, self-reports on this topic are often incomplete and approximate, and they are often influenced by the patient’s memories, mood, and perception. In agreement, among the overall incidence of single-trait frailty indicators (gait speed, grip strength, exhaustion, weight loss, low exercise), weight loss is the worst indicator of frailty, with 8.3% sensitivity [58], thus indicating it as a non-essential indicator in frailty definition. Prospective studies aimed to evaluate the predictive value of frailty development of the modified version FRASNET score are needed. Second, we relied on the GDS-15 questionnaire standardized for the evaluation of depression, which also considers some anxiety symptoms among its items. Regarding stress, in the SF-36, which assesses the quality of life, the mental health index reports the psychological state of patients, thus informing about their self-perceived mental distress. However, other tools could have been valuable for a more detailed assessment, such as CPAP and DASS-21 [59,60]. Finally, the observational nature of FRASNET does not allow for predictive conclusions to be drawn. However, the usage of conditional inference tree analysis leads to a novel data interpretation as smaller subsets that differ significantly in the level of the response variable; thus, the higher the position of the node in the tree shows the more powerful influencing variable on the variability of the response.

## 5. Conclusions

In conclusion, perceptions of QoL, both physical and mental and subclinical depression are major contributors to frailty, with SF-36-PCS scores reflecting self-perceptions of QoL physical health, appearing to be protective against frailty. The results agree well with those of recent longitudinal studies [61] and suggest that the inclusion of simple psychological categorization in the assessment of older adults might have value in patient stratification. Pre-frail subjects represent a particularly interesting group for studies aimed at evaluating whether modifying the identified factors, which are possibly associated with molecular interventions proposed for modulating the inflammatory system, can influence the effective trajectory leading to frailty, as suggested by results in experimental systems [62].

## Figures and Tables

**Table 1:** Characteristics of the FRASNET participants analyzed for frailty.

Parameter	Total = 1204 n (%)	Mean ± SD	Median (Interquartile Range: 25th to 75th Percentiles)
Gender			
Male	481 (40.0)		
Female	723 (60.0)		
Age		73.3 ± 5.73	
Age groups			
65–75 years	815 (67.7)		
76–85 years	349 (29.0)		
>85 years	40 (3.3)		
Weight		70.9 ± 13.02	
BMI		27.0 ± 4.22	
BMI groups			
Underweight (<18.5)	7 (0.6)		
Normal weight (18.5–24)	397 (33.0)		
Overweight (25–29)	541 (44.9)		
Obesity (30–40)	253 (21.0)		
Morbid obesity (>40)	6 (0.5)		
Educational level (n = 1201)			
Primary	201 (16.7)		
Lower secondary	274 (22.8)		
Upper secondary	514 (42.8)		
University	212 (17.7)		
Leisure activities (n = 1186)	906 (75.4)		
Annual income (n = 1187)			
>10,000 euros	1075 (90.6)		
Caregiver (n = 1177)	557 (47.3)		
Living alone (n = 1196)	289 (24.2)		
Number of daily medications (quartiles)			
1 medication	328 (27.3)		
2–3 medications	413 (34.3)		
4–5 medications	275 (22.8)		
>5 medications	188 (15.6)		
Frailty			
Robust	334 (27.7)		
Pre-frail	449 (37.3)		
Frail	421 (35.0)		
SF-36-MCS			51.0 (44–55)
SF-36-PCS			48.0 (41–52)
MMSE			
Normal cognitive functioning (30–24)	1337 (94.4)		
Moderate cognitive functioning (23–18)	67 (5.6)		
GDS-15 (n = 1192)			
Without depression (0–5)	1016 (85.2)		
Mild depression (6–9)	125 (10.5)		
Moderate/Severe depression (10–13)	51 (4.3)		

Values are shown as mean ± standard deviation or median (interquartile range: 25th to 75th percentiles) or n (%). BMI = body mass index; SF-36-MCS = Short Form Survey-36-Mental Component Summary; SF-36-PCS = Short Form Survey-36-Physical Component Summary; MMSE = Mini-Mental State Examination; GDS-15 = Geriatric Depression Scale.

**Table 2:** Summary statistics of significant SNP associations with psychological parameters and with frailty.

**SF-36-PCS** **SNP**	**Risk Allele**	**Genotype**	**Mean (st dev)**		**Comparison ^a^**	**Beta**	**95% CI**	***p* Value**
*CNTF* rs1800169 [G>A]	G	AA AG GG	46.6 (8.3) 47.5 (8.5) 46.1 (8.5)		GG vs. AG/AA	−1.33	[−2.36;−0.30]	0.007
*TGFB1* rs1800469 [C>T]	C	CC CT TT	45.7 (8.5) 47.3 (8.4) 46.6 (8.5)		CC vs. TC/TT	−1.38	[−2.36;−0.40]	0.010
*MTR* rs1050993 [G>A]	G	AA AG GG	47.5 (8.9) 46.3 (8.4) 46.1 (8.3)		AG/GG vs. AA	−1.29	[−2.46;−0.13]	0.019
**MMSE** **SNP**	**Risk Allele**	**Genotype**	**% MMSE < 24**		**Comparison ^b^**	**OR**	**95% CI**	***p* Value**
*IL6* rs1800795 [G>C]	G	CC CG GG	7 3.4 8.9		CG/GG vs. CC	2.26	[1.37;3.79]	0.0016
**Frailty** **SNP**	**Risk Allele**	**Genotype**	**% Pre-Frailty**	**% Frailty**	**Comparison ^c^**	**OR**	**95% CI**	***p* Value**
*FN1* rs7567647 [G>A]	A	AA AG GG	23.1 34.6 39.6	61.5 33.8 34.4	AA vs. AG AA vs. GG	3.85 3.45	[1.92;8.33] [1.75;7.69]	0.0003 0.0006

^a^ Linear regression model with covariates weight groups, daily drugs, presence of caregiver, school years and annual outcome. ^b^ Logistic regression model with covariates sex, weight groups, daily drugs, presence of caregiver, leisure activities and annual outcome. ^c^ Nominal model with covariates sex, age groups, weight groups, daily drugs, presence of caregiver, school years, leisure activities and annual outcome. *p* values are relative to normalized values.

## Data Availability

The data that support the findings of this study are available upon request from the corresponding author.

## Supplementary Materials

The following supporting information can be downloaded at: https://www.mdpi.com/article/10.3390/ijerph192416776/s1, Table S1. Centers of enrollment of FRASNET volunteers (n = 1250) in Milan (northern Italy) and nearby area; Table S2. Descriptive statistics for measurement of cytokines; Table S3. SNPs and allele frequencies among FRASNET participants; Table S4. Linear regression analysis for SF-36-PCS; Table S5. Linear regression analysis for SF-36-MCS; Table S6. Nominal regression analysis for GDS-15; Table S7. Logistic regression analysis for MMSE; Figure S1. Binary tree computed by conditional recursive partitioning of frailty classes (0 = robust, 1 = pre-frail, 2 = frail subjects), including FN1 rs7567647 genetic variant. SF-36-PCS, SF-36-MCS, GDS-15 (0–5 = no depression, 6–9 = mild depression, 10–13 = depression), MMSE, sex, age groups, BMI classes, number of daily drugs (“Drugs”), absence or presence of caregiver (NO/YES), annual income, absence or presence of leisure activities (NO/YES), and level of education (School). The variables affecting frailty are SF-36-PCS, having or not a caregiver, SF-36-MCS, and *FN1* rs7567647 genetic variant.
