# Supplementary material for: Association between Perceived Health-Related Quality of Life and Depression with Frailty in the FRASNET Study
Source: Int J Environ Res Public Health. 2022 Dec 14;19(24):16776. doi: 10.3390/ijerph192416776 (PMC9779617; doi:10.3390/ijerph192416776)
Supplement: Supplementary file 1 [file ijerph-19-16776-s001.zip › ijerph-2029504-supplementary.pdf]

SUPPLEMENTARY MATERIALS

METHODS
Psychometric Measurements
Immunological determinations

Table S1. Centers of enrolment of FRASNET volenteers (n = 1,250) in Milan (northern Italy) and nearby area.

Table S2. Descriptive statistics for measurement of cytokines.

Table S3. SNPs and allele frequencies among FRASNET participants.

Table S4. Linear regression analysis for SF-36-PCS.

Table S5. Linear regression analysis for SF-36-MCS.

Table S6. Nominal regression analysis for GDS-15.

Table S7. Logistic regression analysis for MMSE.

Figure S1. Binary tree computed by conditional recursive partitioning of frailty classes (0 = robust, 1 = pre-frail, 2 = frail subjects), including FN1 rs7567647 genetic variant. SF-36-PCS, SF-36-MCS, GDS-15 (0-5 = no depression, 6-9 = mild depression, 10-13 = depression), MMSE, sex, age groups, BMI classes, number of daily drugs ("Drugs"), absence or presence of caregiver (NO/YES), annual income, absence or presence of leisure activities (NO/YES), and level of education (School). The variables affecting frailty are SF-36-PCS, having or not a caregiver, SF-36-MCS, and *FN1* rs7567647 genetic variant.

METHODS
Psychometric Measurements
SF-36 Medical Outcomes Study Questionnaire Short Form 36 (SF-36) health survey
SF-36 have items that are divided into 8 sub-scales that can be conceptually referred to 8 health domains: Physical activity (AF), Role limitations due to health Physical Pain (PR), Role Limitations Due to Emotional State (ER), Physical Pain (BP), Perception of General Health (GH), Vitality (VT), Social Activities (SF), Mental Health (HD) and a single item on the change in health status.
The physical component summary (Short Form Survey-36-Physical Component Summary, SF-36-PCS) includes four scales: physical functioning (10 items), role-physical (4 items), bodily pain (2 items), and general health (5 items). The mental component summary (Short Form Survey-36-Mental Component Summary, SF-36-MCS) is composed of vitality (4 items), social functioning (2 items), role-emotional (3 items), and mental health (5 items).

Immunological determinations
Level of twenty-five cytokines was measured using a commercially available multiplex bead-based sandwich immunoassay kit (Bio-Plex Pro Human Cytokine 27-Plex Immunoassay, Bio-Rad, Italy) as per the manufacturer's instructions. Briefly, plasma samples (25 μL/well) or standards (25 μL/well) were incubated with 25 μL of the pre-mixed bead sets in pre-wetted 96-well microtiter plates at 4°C overnight. After washing, 25 μL of the fluorescent detection antibody mixture were added for 30 min and 25 μL of streptavidin-phycoerythrin were added to each well for an additional 30 min at room temperature.
In particular, epidermal growth factor (EGF), fibroblast growth factor 2 (FGF2), FMS-like tyrosine kinase 3 ligand (Flt-3L), granulocyte colony-stimulating factor (G-CSF), granulocyte-monocyte colony stimulating factor (GM-CSF), interferon-α2 (IFN-α2), IFN-γ, interleukine (IL)-10, IL-15, IL-17, IL-1β, IL-2, IL-6, IL-8, C-X-C motif chemokine ligand 10 (CXCL10), C-C motif chemokine ligand 2 (CCL2), C-C motif chemokine ligand 4 (CCL4), platelet-derived growth factor-AA (PDGF-AA), soluble CD40 ligand (sCD40L), transforming growth factor alpha (TGF-α), TNF-α and vascular endothelial growth factor (VEGF) were included in the immunoassay kit.

Table S1. Centers of enrolment of FRASNET volunteers (n = 1,250) in Milan (northern Italy) and nearby area.

Center	Location	Period	n
San Raffaele Scientific Institute	Milano	April 2017 - October 2020	631
Università del Tempo Libero Auser	Milano	April 2018	43
RSA Santa Giulia	Milano	May 2018	8
Università del Tempo Libero Auser	Seregno (MB)	May 2018 - January 2019	159
Casa di Riposo Agostoni	Lissone (MB)	January 2019	11
Cuggiono Hospital	Cuggiono (MI)	February 2019	8
Centro Ricreativo Anziani	Gorgonzola (MI)	March 2019 - May 2019	107
Movimento Terza Età	Carugate (MI)	May 2019 - October 2019	63
Residenza Amica	Giussano (MB)	October 2019	40
Ca' Vera	Giussano (MB)	November 2019- January 2020	64
Associazione Pensionati	Pessano con Bornago (MI)	November 2019 - December 2019	53
Centro Anziani	Segrate (MI)	January 2020 - February 2020	63

Notes: MB = Monza e Brianza; MI = Milano.

Table S2. Descriptive statistics for measurement of cytokines.

Cytokine (pg/mL)	N	Means	SD	SE	Minimun	Maximum
Eotaxin	75	80.43	45.93	5.30	26.16	279.07
FGF2	75	30.46	9.34	1.08	11.23	55.12
G-CSF	75	84.19	47.01	5.43	11.76	271.51
IFN-γ	74	4.99	4.07	0.47	0	21.83
IL-1β	75	1.87	1.99	0.23	0.01	10.15
IL-2	75	2.84	1.87	0.22	0	8.81
IL-4	75	6.06	2.09	0.24	2.87	14.47
IL-5	37	10.03	5.78	0.95	1.69	24.60
IL-6	75	3.24	3.98	0.46	0	24.45
IL-7	75	28.71	13.91	1.61	3.51	72.31
IL-8	75	5.95	4.22	0.49	0	22.18
IL-9	75	267.83	77.51	8.95	82.01	504.74
IL-10	37	5.93	3.01	0.50	0.08	16.70
IL-12_p70	37	2.26	1.89	0.31	0.12	7.40
IL-13	75	9.08	8.49	0.98	0	43.93
IL-17	75	8.74	3.45	0.40	3.60	21.50
ILra	75	232.14	108.99	12.58	90.56	747.16
CXCL10	75	667.08	324.98	37.53	232.88	1753.08
CCL2	75	24.16	13.80	1.59	0	56.89
CCL4	75	81.39	19.98	2.31	31.84	137.57
CCL3	75	2.58	1.12	0.13	0.82	7.24
PDGF-bb	75	431.14	422.39	48.77	0	2573.94
CCL5	75	3316.41	2136.42	246.69	546.99	9216.97
TNF-α	75	50.31	20.03	2.31	21.13	110.05
VEGF	75	121.54	84.11	9.71	0	363.26

Notes: fibroblast growth factor 2 (FGF2), granulocyte colony-stimulating factor (G-CSF); interferon γ (IFN-γ); interleukin (IL)-1β, IL-2, IL-4, IL-5, IL-6, IL-7, IL-8, IL-9, IL-10, IL-12_p70, IL-13, IL-17, IL-ra; C-X-C motif chemokine ligand 10 (CXCL10); C-C motif chemokine ligand 2 (CCL2); C-C motif chemokine ligand 3 (CCL3); C-C motif chemokine ligand 4 (CCL4); platelet-derived growth factor-bb (PDGF-bb); C-C motif chemokine ligand 5 (CCL5); tumor necrosis factor (TNF-α); vascular endothelial growth factor (VEGF).

Table S3. SNPs and allele frequencies among FRASNET participants.

SNP	Gene	Chr	position (bp)	Alleles (minor/ major)	MAF	H-W	Reference
rs10883631	*BTRC*	10	101379138	A/G	0.497	0.767	18
rs6747918	*CASP8*	2	201232852	G/A	0.487	0.616	18
rs129968	*CREBBP*	16	3741260	A/G	0.373	0.998	18
rs7567647	*FN1*	2	215432137	A/G	0.195	0.773	18
rs2287396	*GSTZ1*	14	77327849	T/C	0.188	0.746	18
rs2929408	*KAT2B*	3	20041477	T/G	0.226	0.386	18
rs1050993	*MTR*	1	236899005	A/G	0.446	0.387	18
rs1400657	*STAT1*	2	190968814	C/A	0.130	0.237	18
rs2833383	*TIAM1*	21	31343131	T/C	0.274	0.536	18
rs1800169	*CNTF*	11	58624028	A/G	0.166	0.141	19
rs1800795	*IL6*	7	22727026	C/G	0.318	0.672	20
rs1800469	*TGFB1*	19	41354391	T/C	0.363	0.042	21
rs17042917	*IL1RN*	2	113113086	A/G	0.080	0.063	22
rs4251961	*IL1RN*	2	113116890	C/T	0.398	0.138	22
rs3865444	*CD33*	19	51224706	T/G	0.277	0.532	23
rs12552	*OLFM4*	13	53051646	T/C	0.453	0.571	24

Notes: SNPs chromosome positions based on GRCh38 (release 108) assembly. SNP = Single Nucleotide Polymorphism; Chr = chromosome; bp = base pair; MAF = Minor Allele Frequency; H-W = Hardy-Weinberg equilibrium p value.

Table S4. Linear regression analysis for SF-36-PCS.

Parameter	OR	CI 95%	p-value
BMI classes	0.96	[0.83-1.11]	0.598
Daily drugs	1.24	[1.11-1.39]	**1.57E-04**
Caregiver	1.15	[0.92-1.44]	0.211
School years	0.85	[0.75-0.95]	**0.006**
Annual income	1.06	[0.72-1.56]	0.761
SF-36-PCS	0.51	[0.44-0.57]	**7.81E-26**

Notes: BMI = body mass index; SF-36-PCS = Short Form Survey-36-Physical Component Summary.

Table S5. Linear regression analysis for SF-36-MCS.

Parameter	OR	CI 95%	p-value
BMI classes	0.96	[0.83-1.11]	0.591
Daily drugs	1.36	[1.22-1.52]	**3.84E-08**
Caregiver	1.02	[0.82-1.27]	0.851
School years	0.8	[0.71-0.9]	**2.17E-04**
Annual income	0.88	[0.6-1.28]	0.491
SF-36-MCS	0.63	[0.56-0.71]	**7.42E-15**

Notes: BMI = body mass index; SF-36-MCS = Short Form Survey-36-Mental Component Summary.

Table S6. Nominal regression analysis for GDS-15.

Parameter	OR	CI 95%	p-value
Sex	1.08	[0.85-1.36]	0.524
Age groups	1.58	[1.26-1.99]	**6,79E-05**
BMI classes	0.95	[0.82-1.1]	0.481
Daily drugs	1.24	[1.11-1.39]	**2.37E-04**
Caregiver	1.20	[0.96-1.5]	0.117
School years	0.81	[0.72-0.92]	**9.35E-04**
Leisure activities	0.60	[0.46-0.78]	**1.89E-04**
Annual income	1.11	[0.75-1.65]	0.592
GDS-15 classes	2.51	[1.95-3.28]	**4.29E-12**

Notes: GDS-15 = Geriatric Depression Scale.

Table S7. Logistic regression analysis for MMSE.

Parameter	OR	CI 95%	p-value
Sex	1.22	[0.98-1.53]	0.081
BMI classes	0.96	[0.84-1.11]	0.604
Daily drugs	1.39	[1.25-1.55]	**1.61E-09**
Caregiver	1.14	[0.92-1.42]	0.238
Leisure activities	0.57	[0.44-0.74]	**2.13E-05**
Annual income	0.87	[0.6-1.26]	0.456
MMSE classes	0.56	[0.35-0.89]	**0.014**

Notes: MMSE = Mini-Mental State Examination

Figure S1. Binary tree computed by conditional recursive partitioning of frailty classes (0 = robust, 1 = pre-frail, 2 = frail subjects), including FN1 rs7567647 genetic variant.

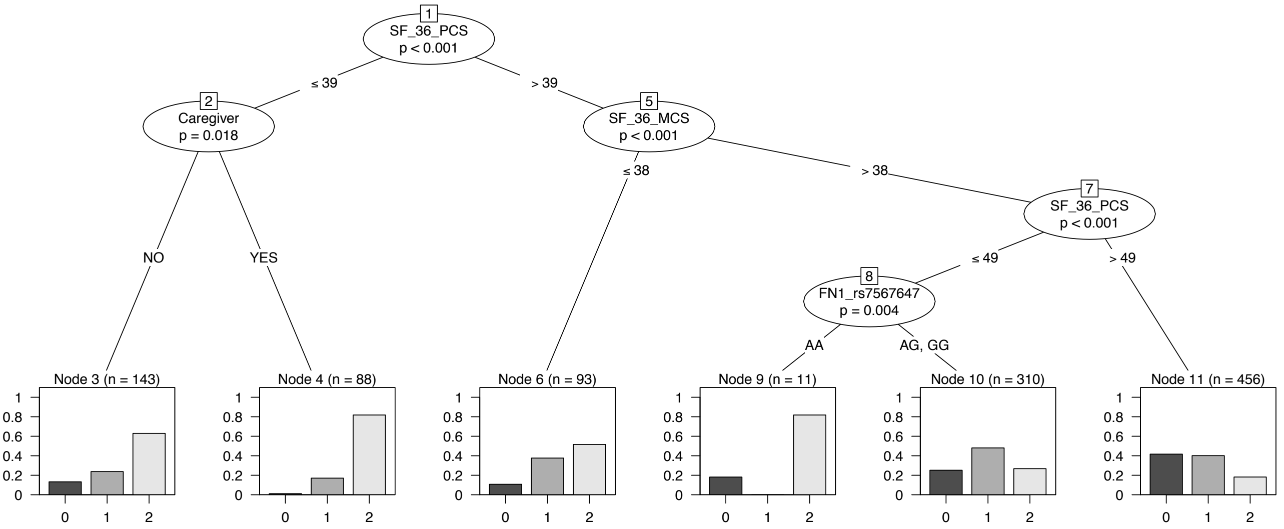

SF-36-PCS, SF-36-MCS, GDS-15 (0-5 = no depression, 6-9 = mild depression, 10-13 = depression), MMSE, sex, age groups, BMI classes, number of daily drugs ("Drugs"), absence or presence of caregiver (NO/YES), annual income, absence or presence of leisure activities (NO/YES), and level of education (School).
